# Trypanocidal Activity of Thioamide-Substituted Imidazoquinolinone: Electrochemical Properties and Biological Effects

**DOI:** 10.1155/2013/945953

**Published:** 2013-07-08

**Authors:** Fernanda M. Frank, Alejandra B. Ciccarelli, Mariela Bollini, Ana M. Bruno, Alcira Batlle, Maria E. Lombardo

**Affiliations:** ^1^Departamento de Microbiología, Facultad de Medicina, IMPaM (UBA-CONICET) and Cátedra de Inmunología, Facultad de Farmacia y Bioquímica, UBA, Paraguay 2155 P13, 1121 Buenos Aires, Argentina; ^2^Centro de Investigaciones Sobre Porfirinas y Porfirias, CIPYP (UBA-CONICET), Hospital de Clínicas José de San Martín, UBA, Córdoba 2351, 1120 Buenos Aires, Argentina; ^3^Departamento de Química Orgánica, Facultad de Farmacia y Bioquímica, UBA, Junín 956, 1113 Buenos Aires, Argentina; ^4^Departamento de Química Biológica, Facultad de Ciencias Exactas y Naturales, 2° Piso CM1, UBA, Int. Güiraldes 2160, CABA, 1428 Buenos Aires, Argentina

## Abstract

Three thioamide-substituted imidazoquinolinone, which possess a heterocyclic center similar to tryptanthrin and are named **C1**, **C2**, and **C3**, were studied regarding (a) their *in vitro* anti-*Trypanosoma cruzi* activity, (b) their cytotoxicity and electrochemical behaviour, and (c) their effect on cell viability, redox state, and mitochondrial function. The assayed compounds showed a significant activity against the proliferative forms, but only **C1** showed activity on the trypomastigote form (for **C1**, IC_50  epi_ = 1.49 *μ*M; IC_50  amas_ = 1.74 *μ*M; and IC_50  try_ = 34.89 *μ*M). The presence of an antioxidant compound such as ascorbic acid or dithiotreitol induced a threefold increase in the antiparasitic activity, whereas glutathione had a dual effect depending on its concentration. Our results indicate that these compounds, which exhibited low toxicity to the host cells, can be reduced inside the parasite by means of the pool of low molecular weight thiols, causing oxidative stress and parasite death by apoptosis. The antiparasitic activity of the compounds studied could be explained by a loss of the capacity of the antioxidant defense system of the parasite to keep its intracellular redox state. **C1** could be considered a good candidate for *in vivo* evaluation.

## 1. Introduction

Chagas' disease is endemic in Latin America, directly affecting around 20 million of inhabitants, but it is also to be taken in account that more than 200 million are at risk of infection [1]. *Trypanosoma cruzi* is the etiological agent of this illness, a hemoflagellate parasite whose life cycle involves the obligatory passage through both vertebrate and invertebrate hosts (hematophagous triatomine bugs). Currently, only two drugs were commercially available for its treatment: Benznidazole (Bnz) and Nifurtimox (Nfx); however, the latter has been discontinued. Both drugs can reduce serological titters in acute and early chronic infections by eliminating patent parasitemia. Nevertheless, these drugs are not active against all *T. cruzi* strains and are known to produce toxic effects on the host [2]. All of these facts highlight the urgent need for the development of new, cheap, safe, and more efficient compounds for treating Chagas' disease.

**Figure 1:**
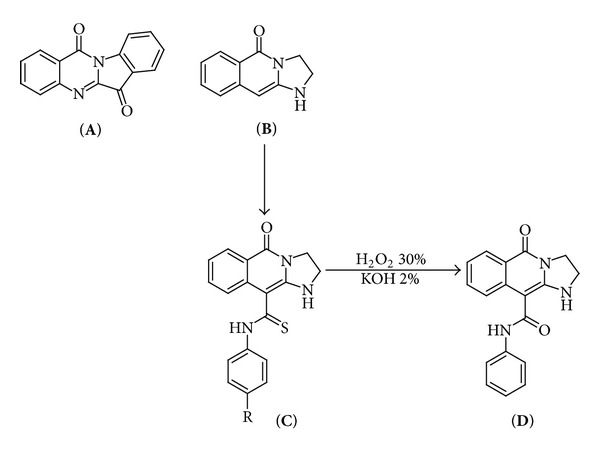
Tryptanthrin and structurally related compounds. (**A**) Tryptanthrin. (**B**) heterocycle precursor. (**C**) C-10 thioamide-substituted compounds, denominated **C1** (R: H), **C2** (R: Cl), and **C3** (R: CH_3_). (**D**) C-10 amide-substituted compound.

A variety of natural products are known to have antitrypanosomal activity. Tryptanthrin (indolo[2,1-b]quinazoline-6,12-dione) is a weak basic alkaloid isolated from medicinal plants, such as *Polygonum tinctorium*, *Isatis indigotica*, and *Strobilanthes cusia* [3]. This alkaloid has a broad spectrum of biological functions, including anti-inflammatory, antifungal, antibacterial, and antitumor effects [4] and its references. In addition, tryptanthrin derivatives have been shown to possess activity against several protozoan pathogens. Specifically, these compounds have been reported to inhibit some strains of *Leishmania* spp. [5, 6], *Trypanosoma brucei* [7], *Plasmodium falciparum* [8], and *Toxoplasma gondii* [9]. Closely related to the tryptanthrin structure (Figure 1(**A**)), we have synthesized the 2,3-dihydroimidazo[1,2-b]isoquinolin-5(1-*H*)-one molecule, which is a heterocycle precursor (Figure 1(**B**)) to then obtain new N-1 or C-10 substituted imidazoquinolinones derivatives, similar to tryptanthrin [10, 11]. Importantly, when a computational analysis was run over, comparing tryptanthrin and the heterocycle precursor structures, which consist in the overlapped structures (HyperChem software, Hypercube. Inc), a significantly great similarity was found (root-mean-square deviation, RMSD 1.69 × 10^−3^ Å; unpublished data). Recalling that thioamide derivatives substituted in C-10 (Figure 1(**C**)) exhibited the best antichagasic activity on *T. cruzi *epimastigotes [11], we have therefore selected the compounds of the **C** series (**C1**, **C2**, and **C3**, Figure 1), which showed superior activity to that of Nfx, to study their effect *in vitro *against the different stages of the parasite. **D** was used as a non-C-10 thioamide substituted imidazoquinolinone derivative (Figure 1(**D**)). The mechanism of action of the **C** compounds was also investigated. In case that **C** compounds were efficient antichagasic agents, it is worth to emphasize that they present the additional advantages over tryptanthrin and its derivatives of being obtained more cheaply, simply, and efficiently.

## 2. Materials and Methods

### 2.1. Chemicals

Bnz was kindly provided by Roche (Argentina). The compounds assayed (Figure 1) N-phenyl-5-oxo-1,2,3,5-tetrahydroimidazo[1,2-b]isoquinolin-10-carbothioamide (**C1**), N-(4-chlorophenyl)-5-oxo-1,2,3,5-tetrahydroimidazo[1,2-b]isoquinolin-10-carbothioamide (**C2**), N-(4-methylphenyl-5-oxo-1,2,3,5-tetrahydroimidazo[1,2-b]isoquinolin-10-carbothioamide (**C3**), and 5-oxo-N-phenyl-1,2,3,5-tetrahydroimidazo[1,2-b]isoquinolin-10-carboxamide (**D**) were synthesized according to Bollini et al. [11]. Standard solutions of these compounds were prepared in dimethyl sulphoxide (DMSO), and their final concentrations in the experiments never exceeded 0.5%.

### 2.2. Parasites

*Trypanosoma cruzi *epimastigotes (Tulahuen strain) were grown as previously described [12]. Bloodstream trypomastigotes were obtained from infected CF1 mice by cardiac puncture. Tulahuen strain expressing the *β*-galactosidase gene was kindly provided by Dr Buckner (University of Washington, USA) [13].

### 2.3. Animals

Inbred CF1 male mice were nursed at Facultad de Medicina, Universidad de Buenos Aires. Animals were handled in accordance with the guidelines established by the Animal Care and Use Committee of the Argentine Association of Specialists in Laboratory Animals (AADEALC).

### 2.4. *In Vitro* Assays for Anti-*T. cruzi* Activity

To evaluate the growth inhibition of epimastigotes, 1.5 × 10^6^ parasites/mL were cultivated with different concentrations of the compounds (0.50 to 15 *μ*M) or Bnz (2.50 to 15 *μ*M) for 4 days. Cells growth was assessed by counting the number of cells per mL of culture using a Neubauer chamber and was expressed as cellular density (CD). The percentage of inhibition (%*I*) was calculated as %*I* = {1 − [(CD_4t_ − CD_0_)/(CD_4c_ − CD_0_)]} × 100, where the different CDs represent the cellular density of CD_4t_, treated parasites on day 4; CD_0_, parasites on day 0; and CD_4c_, untreated parasites (control) on day 4.

The trypanocidal effects of **C1**, **C2**, **C3**, **D** and Bnz were also tested on bloodstream trypomastigotes according to a standard WHO protocol slightly modified [14]. Briefly, mouse blood containing trypomastigotes was treated with different concentrations of each compound (0.30 to 350 *μ*M) or Bnz (0.38 to 38 *μ*M). Plates were incubated for 24 h, and surviving parasites were counted in a Neubauer chamber as previously described [15]. Results were expressed as the percentage of lysed parasites (%*L*) relative to the number of parasites in the control: %*L* = [1 − (CD_t_/(CD_c_)] × 100, where CD_t_ and CD_c_ represent the cellular density of treated and untreated parasites, respectively.

For analysis of amastigotes, J774 cells were infected with bloodstream trypomastigotes expressing the *β*-galactosidase gene at a parasite: cell ratio of 10 : 1. After 24 h, cell cultures were washed and each compound (2 to 100 *μ*M) was added in fresh RPMI medium without phenol red (to avoid interference with absorbance readings at 570 nm). After 7 days, the assay was developed as described [15]. The galactosidase activity was quantified using CPRG as substrate and measuring absorbance at 570 nm in a Microplate Reader (Bio-Rad Laboratories). Since the assayed compounds are coloured, blanks including uninfected cells with different doses of each compound were performed. The percentage of inhibition was calculated as %*I* = {1 − [(*A*_it_ − *A*_nit_)/(*A*_ic_ − *A*_nic_)]} × 100, where *A* represents the mean *A*_570_ value recorded for *A*_it_, treated infected cells; *A*_nit_, treated noninfected cells; *A*_ic_, untreated infected cells; and *A*_nic_, untreated noninfected cells.

### 2.5. Cytotoxicity Assay

Vero cells were cultivated with different concentrations of each compound (12.5–100.0 *μ*M) or Bnz (3.0–3000 *μ*M). After 48 h of incubation, cells were washed and viability was measured by the MTT assay as previously described [15]. The selectivity index (SI) was calculated as the 50% cytotoxic concentration (CC_50_) obtained with Vero cells divided by the 50% inhibitory concentration (IC_50_) obtained with *T. cruzi*.

### 2.6. Electrochemical Behaviour of Thioamide-Substituted Imidazoquinolinones

Cyclic voltammograms for **C1**, **C2**, and **C3** dissolved in 1% DMSO were carried out using an EQMAT instrument with an EQSOFT Processor, at a sweep rate of 0.2 V/s under a nitrogen atmosphere at room temperature, employing lithium perchlorate as supporting electrolyte. A three-electrode cell was used equipped with a vitreous carbon as working electrode, a gold wire as auxiliary electrode, and a saturated calomel as reference electrode.

### 2.7. Biochemical Assays to Characterize the Antitrypanosomal Action

Epimastigotes of *T. cruzi* from a 4 days culture were incubated with **C1** (7.5–22.5 *μ*M) during 5–36 hours. Cells were harvested, washed and then the following biochemical assays were carried out.

#### 2.7.1. Evaluation of Oxidative Stress

*(a) Assay of Intracellular Oxidative Activity.* The intracellular oxidative activity was assessed by flow cytometry using the oxidant-sensitive fluorescent probe H_2_DCFDA. As a positive control cells were treated with 0.1 mM H_2_O_2_. Stained cells were then analyzed by a FACSCalibur flow cytometer (Becton Dickinson) with an excitation wavelength of 480 nm and an emission wavelength of 530 nm. Flow cytometry results were expressed by the ratio Gm_t_/Gm_c_, where Gm_t_ and Gm_c_ correspond to the geometric mean of histograms obtained for treated and untreated cells, respectively.

*(b) Determination of Antioxidant Enzymes Activity. *The activities of superoxide dismutase (SOD), ascorbate peroxidase (APx), and trypanothione reductase (TryR) were assayed as previously established [15].

Protein concentration was determined according to the method described by Lowry et al. [16]. These values were used to express the specific enzymatic activities as activity per mg of protein.

*(c) Determination of Total Thiol Groups. *The thiol groups content was determined employing the chromogenic compound 5,5′-dithiobis-2-nitrobenzoate (DTNB), as already described [12].

#### 2.7.2. Evaluation of Parasite Death

Cell viability and phosphatidylserine (PS) exposure on the parasite surface were assessed by propidium iodide (PI) and Annexin V-fluorescein isothiocyanate (FITC) staining, according to the manufacturer's instructions (Invitrogen). Epimastigotes exposed to 30% fresh human serum for 2 h at 28°C were used as positive control. Parasites were analysed by flow cytometry acquiring 20,000 events per sample.

#### 2.7.3. Evaluation of Mitochondrial Damage

Mitochondrial membrane potential was assessed using two well-established assays: 3,3′-dihexyloxacarbocyanine iodide (DiOC_6_) staining and the cytochrome c release.

For the DiOC_6_ assay, epimastigotes (10^6^) were permeabilized for 20 min at room temperature with 0.01% saponin, washed, and incubated with 30 nM DiOC_6_ for 30 min at 37°C. The positive control was done with 250 nM trifluoromethoxy carbonyl cyanide phenyl hydrazone (FCCP) as depolarizing agent. Stained cells were analyzed by flow cytometry with an excitation wavelength of 484 nm and an emission wavelength of 511 nm.

To evaluate the cytochrome c release, parasites (3 × 10^8^) were resuspended in PBS containing 200 *μ*g/mL digitonin, incubated on ice for 15 min and then centrifuged at 9 000 ×g for 10 min at 4°C. Both, mitochondrial-rich fraction (pellet, resuspended in 50 *μ*L of PBS) and cytosolic fraction (150 *μ*L) were subjected to Western-blot analysis for cytochrome c. Protein extracts (10 *μ*L and 30 *μ*L of mitochondrial and cytosolic fractions, resp.) were resolved by 14% SDS/PAGE, transferred to nitrocellulose membranes, blocked with 3% skimmed milk in PBS, and then incubated with specific antibodies, according to the protocol of Piacenza et al. [17]. Relative intensities of bands were quantified by densitometry using Scion Image software (Scion). Results were expressed in arbitrary units.

### 2.8. Statistical Analysis

The results presented are representative of three to four separate experiments, performed in duplicates or triplicates. All data are expressed as means ± standard errors of the mean (SEM). To calculate the IC_50_ values, the %*I* or %*L* values were plotted against the log of drug concentration (*μ*M) and fitted with a straight line determined by a linear regression (Sigma Plot 10 software). The significance of differences was evaluated using Student' *t* test, taking a *P* < 0.05 as significant. Flow cytometry data were analyzed employing the WinMDI 2.9 software.

## 3. Results

### 3.1. *In Vitro* Antitrypanosomal Activity

Results for the *in vitro* assays against the different stages of the parasite are shown in Table 1. Even though the three compounds of the series **C** were found to be active, with very similar IC_50_ values for epi- and amastigotes, on trypomastigotes **C1** was 10 times more active than **C2** and **C3**. All the tested compounds showed the lowest *in vitro* trypanocidal activity when evaluated on the trypomastigote forms. Comparing the values obtained for **C** compounds and **D** (C-10 amide substituted analogue) it can be concluded that the thioamide group is essential for drugs to have a considerable anti-*T. cruzi* activity. Because of these findings, **C1** was selected as the most active compound of the **C** series, rendering the highest antiparasitic activity against the three stages of *T. cruzi*. Notably, although IC_50_ of **C1** and Bnz were similar on trypomastigote stage, **C1** was found to be 3.5 times more active than Bnz on the epimastigote form (Table 1).

### 3.2. Cytotoxicity Assay

Unlike Bnz, which displayed a CC_50_ of 82.79 ± 2.75 *μ*M, none of the compounds evaluated were cytotoxic at any of the concentration assayed (12.5–100.0 *μ*M). Since all the compounds displayed a CC_50_ greater than 100.0 *μ*M, then the highest SI was obtained for drugs that had the lowest IC_50_ value (Table 1). **C1** was the compound presenting good SI values for the three stages. 

### 3.3. Electrochemical Behaviour

**Figure 2:**
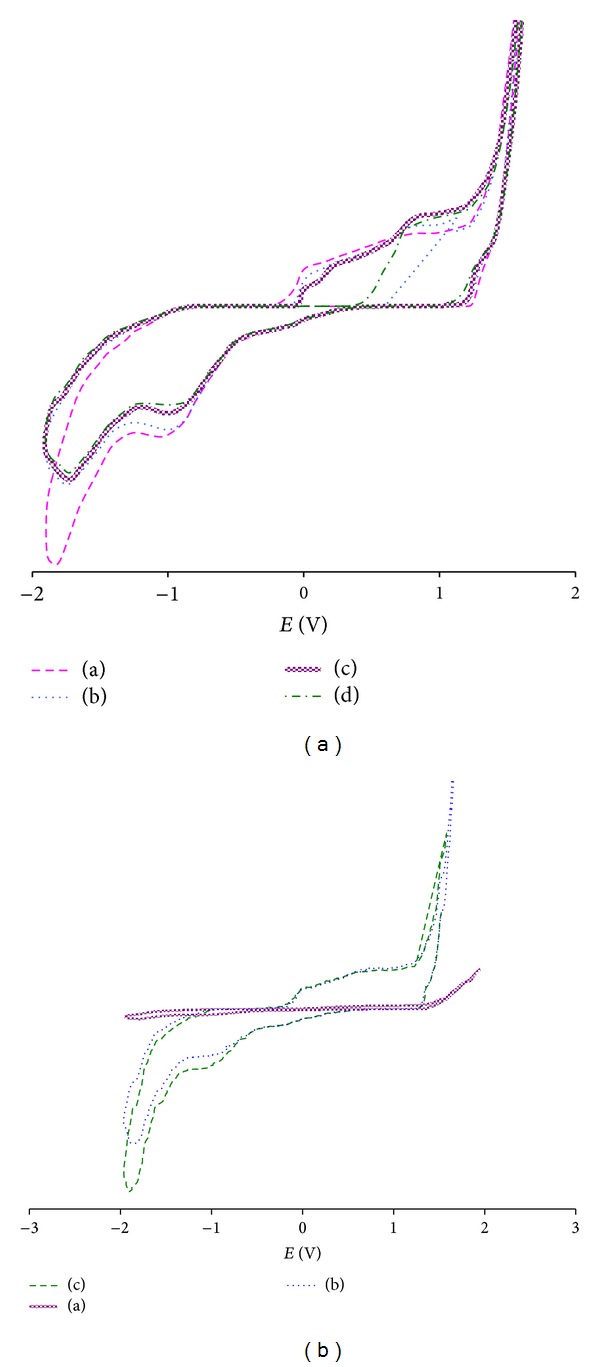
Cyclic voltammograms of **C1**. Profiles corresponding to: (A) different concentrations of drug in 1% DMSO: (a) 0.014 *μ*M; (b) 0.14 *μ*M; (c) 0.28 *μ*M; and (d) 0.90 *μ*M. (B) (a) **C1**; (b) GSH: **C1** (1 : 1); and (c) GSH: **C1** (2 : 1). Experimental conditions were as described in Section 2.

Cyclic voltammetry is a methodology extensively used to determinate redox properties of molecules in solution.  Figure 2(A) shows the cyclic voltammograms for increasing amounts of compound **C1** in 1% DMSO. A cathodic peak at −1.04 V and an anodic peak at 0.78 V, corresponding to irreversible reactions of reduction and oxidation, respectively, were observed. A very good linear correlation between the cathodic peak current (*i*_cp_) and concentration of **C1** from 0.014 to 0.28 mM was observed (graphic not shown). For higher drug concentrations (0.90 mM) the saturation of the electrode was evident. A similar behaviour was obtained for the other two compounds of the **C** series (data not shown). Neither the cathodic nor the anodic peaks were measurable for the heterocycle precursor (Figure 1(**B**)) when subjected to the same potentials as **C1**.

The electrochemical properties of **C1** in the presence of a biologically relevant thiol, glutathione (GSH), were also studied.  Figure 2(B) shows the typical cycle voltammograms of **C1** in the absence and in the presence of increasing amounts of GSH. When GSH was added at the ratio GSH : **C1** (1 : 1) it produced a significant increase in the current of both anodic and cathodic peaks with a concomitant displacement of the cathodic peak to low potentials. At the ratio GSH : **C1** (2 : 1) only the cathodic peak was increased, whereas the anodic peak was the same as that obtained with the 1 : 1 ratio. The GSH signals, at the studied concentrations, did not interfere with the signals of **C1**. The electrochemical profile obtained by adding GSH suggests that new electroactive entities (oxidation and reduction products) had been generated. The presence of a adduct **C1**-GSH was supported by the remarkable increase in the cathodic peak current with a concomitant displacement to lower potentials.

**Figure 3:**
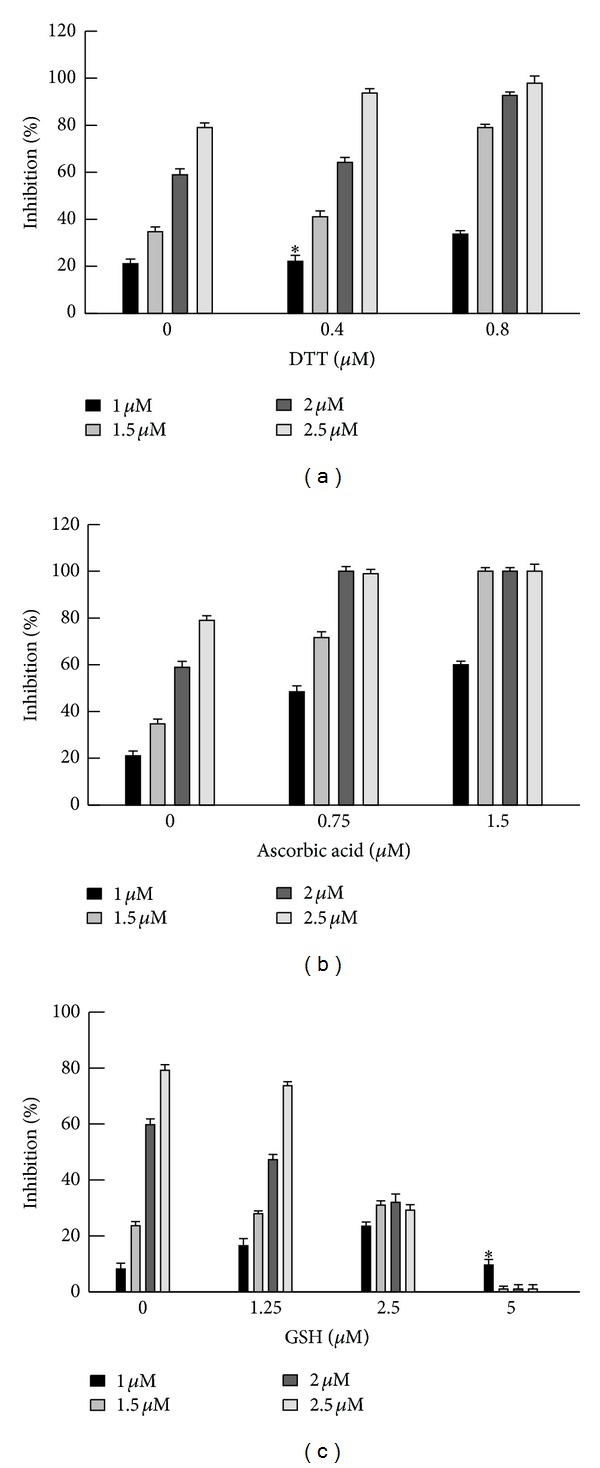
Effect of DTT, ascorbic acid and GSH on the anti-*T. cruzi* activity of **C1**. Experimental conditions were as described in Section 2. The concentrations of **C1** tested were from 1 up to 2.5 *μ*M. The value 0% of inhibition corresponds to parasites cultured in the absence of both **C1** and antioxidant compound. *No significant differences (*P* > 0.05) were found when compared to the control (0 *μ*M antioxidant compound) as assessed by Student' *t* test.

To obtain additional information regarding the mechanism of action of **C** compounds, the effect of antioxidant agents, such as dithiothreitol (DTT), ascorbic acid, and GSH on *in vitro* anti-*T. cruzi* activity of **C1** was evaluated (Figure 3). At the concentrations tested both DTT and ascorbic acid considerably enhanced the inhibitory action of the drug. Higher concentrations of these antioxidants were not tested because *in vitro* they showed significant antiparasitic activity *per se*. For GSH, a dual effect was observed; this compound was able to either enhance or inhibit the effect of **C1**, depending on the concentration employed. GSH concentrations up to 2.5 *μ*M potentiated the effect of low concentrations of **C1** (no greater than 1.5 *μ*M), whereas the effect of **C1** concentrations above 1.5 *μ*M was diminished. At a concentration of GSH of 5 *μ*M the inhibitory effect of **C1** was completely abrogated.

### 3.4. Biochemical Assays to Characterize the Antitrypanosomal Action

#### 3.4.1. Evaluation of Oxidative Stress

For this study, epimastigotes are cultured in the presence of high concentrations of **C1** (7.5, 15, and 22.5 *μ*M) during short times of exposure (5, 12, and 24 h). These concentrations and times were selected because they were suitable for the parasite to manifest a response but without causing its death nor/or allowing it to revert such response. According to their electrochemical properties, we expected that **C1** would act as an electrophilic compound able to generate oxidative stress inside the parasite. The fluorescence of H_2_DCFDA-loaded epimastigotes was not significantly modified by **C1** treatment. Thus, the ratio Gm_t_/Gm_c_ was around 1 independently on **C1** concentration and the time of exposure (data not shown). For the same treatment conditions, the activities of SOD and APx (Table 2) were not significantly different from those obtained with the control (only for SOD at longer times and high drug concentrations a slight decline in activity was observed), whereas TryR activity values showed a significant increase for all concentrations of **C1** and all exposure times tested (Table 2). Simultaneously, the level of low molecular mass thiols was found to remain constant for all **C1** concentrations tested, at exposition times of 5 and 12 h (data not shown). After 24 h of incubation a slight decrease (not more than 20%) was only observed for the highest concentration of the drug (22.5 *μ*M). This behaviour, similar to that showed with SOD activity, would indicate that the deleterious action of 22.5 *μ*M **C1** begins to be evident within 24 h of treatment. The constant level of thiols was not surprising due to the high activity of TryR.

#### 3.4.2. Evaluation of Cell Death and Mitochondrial Damage

**Figure 4:**
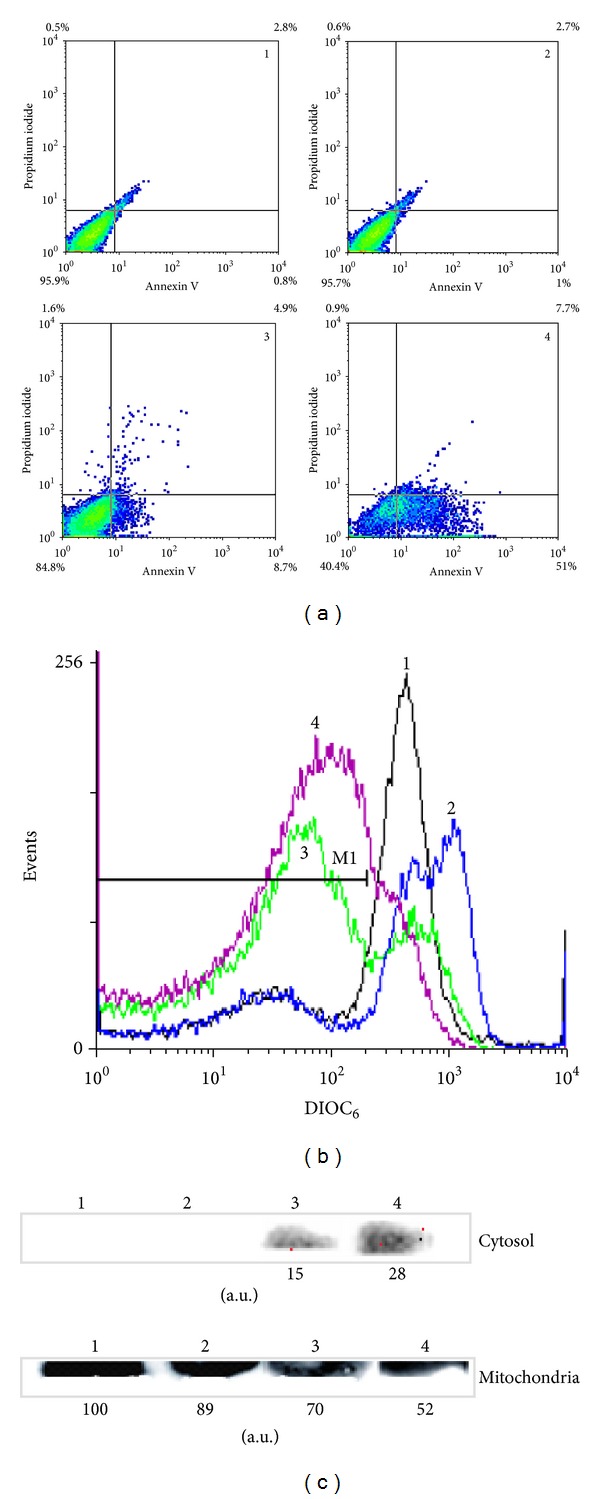
Effect of **C1** on cell death and state of the mitochondria evaluated by flow cytometry and Western blot. Epimastigotes of *T. cruzi* were treated with **C1** 22.5 *μ*M during 8, 24, and 36 h. Parasites were (a) stained by Annexin V-FITC/PI, (b) stained by DIOC_6_, or (c) incubated with digitonin, to obtain the cytosolic and mitochondrial fractions, which were subjected to Western-blot analysis for cytochrome c. The numbers correspond to 1: untreated cells and 2, 3, and 4: treated cells with **C1** during 8, 24, and 36 h, respectively. Methodology and data analysis were carried out as described in Section 2.

For these assays, parasites treated with **C1** at 22.5 *μ*M for 8, 24, and 36 h were used. These experimental conditions were known to lead to parasite death. Annexin-V FITC/PI staining was used as a parameter to detect apoptotic cells. Results demonstrated that the number of apoptotic cells increased during the treatment with **C1** in a time-dependent manner (Figure 4(a)). The most significant differences in the levels of apoptotic cells were observed for early apoptotic cells for which values of 0.8%, 8.7%, and 51.0% were obtained for 0, 24, and 36 h of treatment, respectively. The number of late apoptotic cells also increased with time of treatment but in a less marked and significant manner, obtaining values of 2.8%, 4.9% and 7.7% for 0, 24, and 36 h of treatment, respectively. The number of viable nonapoptotic cells reached values of 84.8% and 40.4% for 24 and 36 h of incubation, respectively, versus 95.9% for control (0 h).

Simultaneously, the mitochondrial membrane depolarization which was evident after 24 h of treatment remained at similar levels up to 36 h (Figure 4(b)). Depolarized cells reached values of 77% and 84% of the evaluated cells after 24 and 36 h of treatment, respectively, versus 31% for the control (0 h). Given that the depolarization of the outer mitochondrial membrane causes the release of cytochrome c into the cytoplasm, then, the presence of cytochrome c in both mitochondrial and cytosolic fractions was assessed (Figure 4(c)). Cytochrome c was detected in the parasite cytosol after 24 h of treatment with **C1** increasing up to 2.5–3 times at 36 h. Densitometric analysis of the immunoblots showed that the total cytochrome c (mitochondrial + released into cytosol) in **C1**-treated parasites remained constant (the mitochondrial fraction of parasites nontreated was considered as control).

## 4. Discussion

In this work we have studied, on all stages of *Trypanosoma *cruzi, the trypanosomal activity of three totally synthetic C-10 thioamide substituted imidazoquinolinones, named **C1**, **C2**, and **C3** (Figure 1(**C**)). As illustrated in Table 1, the three compounds showed a considerable activity against epimastigotes and amastigotes, whereas **C1** (selected as the best antichagasic compound) was the only one displaying an activity value similar to that obtained with Bnz for the infective form. The high IC_50_ values observed for all tested imidazoquinolinones on trypomastigotes could be due to drug instability in presence of whole blood and/or to their association with serum components. Only tested on epimastigotes, the heterocycle precursor did not show antichagasic activity (IC_50_ higher than 25 *μ*M, data not shown). This result together with those obtained for compounds **C** on epimastigotes (Table 1) are consistent with slight differences due to the different methodology used, to the values previously reported by Bollini et al. [11]. The derivatives showed activities between 5 (for **C2**) to 25 (for **C1** and **C3**) times greater than the heterocyclic precursor. Regarding this, the heterocycle precursor and **C** series compounds show on *T. cruzi* a behaviour similar to tryptanthrin and its derivatives on *T. brucei* where the most potent derivative had a 50% effective concentration more than 25 times lower than that of tryptanthrin [11].

From the electrochemical study we would conclude that the reduction of **C** compounds (cathodic peak near −1.04 V) could take place *in vivo*. The absence of the correspondent anodic peak would indicate that this electronic transfer process is irreversible. The anodic scans showed a peak at 0.78 V that would correspond to an irreversible oxidation reaction. Considering that no redox reactivity was measurable for the heterocycle precursor (Figure 1(**B**)) when subjected to the same potentials as **C1**, it can be expected that the reduction of the thioamide group in C-10 could take place in the biological environment and represent a key event in the mechanism of action of these drugs. The reactions (1) and (2) shown below could justify the presence of the two peaks mentioned previously. Because we have found that the cathodic peak current is directly proportional to the **C1** concentrations (Randles-Sevcik equation) then, cyclic voltammetry can be used to quantify concentrations of compounds **C** between 0.014 to 0.28 mM.

On the other hand, the electrochemical profile obtained with GSH : **C1** (1 : 1) suggests that new reduction (cathodic peak) and oxidation (anodic peak) reactions have taken place. Reactions (3) and (4) would justify the cathodic and anodic peaks, respectively. The presence of the **C1**-GSH adduct was supported by both the increase in current and the shift of the cathodic peak to lower potentials. Reaction (4) reaches the saturation when the ratio GSH : **C1** is higher than 1 : 1,

(1)  RNH–R′C=S+2H++2e−→RNH–R′CH–SH

(2)  2RNH–R′CH–SH→RNH–R′CH–S–S–HCR′–HNR+2H++2e−

(3)  RNH–R′CH–SH+GSH→Adduct[RNH–R′CH–SH−(GSH)]

(4)  RNH–R′CH–SH+GSH→RNH–R′CH–S–S–G+2H++2e−

R: −C_6_H_5_ (for **C1**). R′: anion of precursor heterocycle (Figure 1(**B**)).

Considering that trypanosomatids possess high levels of low molecular mass thiols, we could expect that the reaction (2) was not physiologically significant. Then the probable mechanism of action of **C** compounds could be represented by the reactions (1), (3), and (4). It is important to remember that the only low molecular mass thiol of *T. cruzi* is not glutathione because this parasite has significant amounts of trypanothione (N^1^, N^8^ bis glutathionyl-spermidine), glutathionylspermidine, and ovothiol A [18]. Therefore, GSH would not be the only species to accomplish reactions (3) and (4) within the parasite. Independently of the interaction between the reduced compound and low molecular thiols, the possibility of an interaction with essential thiols from parasite's proteins (as enzymes) should be considered. The stimulatory effect of trypanosomal activity of **C1** observed by adding antioxidants such as DTT, ascorbic acid, or low concentrations of GSH (Figure 3) would support the participation of reaction (1) as part of the mechanism of action of this drug. Since the presence of high concentrations of GSH abolishes the antiparasitic effect of **C1**, we could postulate that reactions (3) and (4) would be involved in the metabolism of the drug inside the parasite. The latter reactions would block its effect.

According to these results, **C1** could act as an electrophilic compound, and therefore it could be able of producing oxidative stress inside the parasite. Using **C1** concentrations (7.5–22.5 *μ*M) and exposure times (5–24 h) for which most of the cells remain viable (Figure 4(a)), the intracellular oxidative state, SOD and APx activities, and the levels of –SH groups remained unchanged. On the other hand, the only parameter that was significantly increased even for the lower concentration and shorter treatment time was the activity of TryR (Table 2). An oxidation and/or a decrease in the levels of thiols may have occurred (according to the reactions mentioned previously) as a consequence of the addition of **C1**, but the level of –SH groups was not altered, whereas it may be restored by the TryR.

Finally, we have found that treatment with **C1** (22.5 *μ*M) at times up to 36 h produced time-dependent changes in the mitochondrial membrane potential, cytochrome c release from mitochondria into the cytoplasm, and the exposure of PS on the outer surface leaflet of the plasma membrane (Figure 4). These results would suggest that **C1** induces the parasite death by apoptosis.

## 5. Conclusions

Our findings led us to postulate that (1) to exert their effect, the thioamide-substituted imidazoquinolinones must undergo reduction inside the parasite, (2) the target of these drugs would be the pool of low molecular weight thiols, considering the principal redox buffer in the parasitic protozoa [19], and (3) the antiparasitic activity of these drugs may be associated with a loss of the antioxidant defense system capacity of the parasite to keep the intracellular redox state; however, as it has been reported for other drugs, the activity of C compounds could also be associated with a dysfunction of the only mitochondria of these organisms (concerning this regard, additional studies are necessary to further sustained this proposed) mediated by the loss of mitochondrial membrane potential [20]. Both stress oxidative or mitochondrial damage finally would lead to cell death by apoptosis. These results provide supporting evidence to test *in vivo* the trypanocidal action of **C1** in an animal model of Chagas' disease. Considering the low income of the population suffering from Chagas' disease, it is important to take in account that the synthesis of imidazoquinolinones is highly efficient, simple, fast, and inexpensive; moreover, final products do show good stability. The previous reasons convert these compounds to an attractive therapeutic alternative more interesting than using tryptanthrin or its derivatives, to fight this parasitosis.

## Figures and Tables

**Table 1:** Values of IC_50_ for the activity of the imidazoquinolinones on epi-, trypo-, and amastigotes forms of *Trypanosoma cruzi. *

Compound	Epimastigotes		Trypomastigotes		Amastigotes	
	IC_50_ (*μ*M)	SI	IC_50_ (*μ*M)	SI	IC_50_ (*μ*M)	SI
**C1**	1.49 ± 0.28	>67.1	34.89 ± 1.20	>2.9	1.74 ± 0.30	>57.5
**C2**	5.57 ± 0.53	>18.0	306 ± 15	>0.3	3.63 ± 0.51	>27.5
**C3**	1.50 ± 0.30	>66.7	246 ± 18	>0.4	1.50 ± 0.32	>66.7
**D**	>25	ND	≫300	ND	11.44 ± 1.21	ND
Bnz	5.49 ± 0.89	15.1	30.26 ± 2.85	2.7	ND	ND

IC_50_ and SI values were calculated as indicated in Section 2. ND: not determined.

**Table 2:** Effect of treatment with **C1** on antioxidant enzymes activities.

Time of treatment (hours)	Drug (*µ*M)	SOD activity (%)	APx activity (%)	TryR activity (%)
5	0	100.00 ± 3.60	100.00 ± 3.89	100.00 ± 4.56
	7.5	95.85 ± 2.50	111.26 ± 7.60	152.23 ± 6.38*
	15.0	99.31 ± 4.30	98.31 ± 2.10	177.17 ± 15.56*
	22.5	90.09 ± 2.30	105.20 ± 6.20	143.89 ± 4.80*

12	0	100.00 ± 4.20	100.00 ± 1.50	100.00 ± 8.30
	7.5	99.75 ± 2.90	98.30 ± 2.30	188.60 ± 12.30*
	15.0	103.92 ± 4.30	95.91 ± 4.10	251.62 ± 15.56*
	22.5	88.48 ± 3.20*	93.48 ± 6.20	190.55 ± 14.29*

24	0	100.00 ± 4.50	100.00 ± 4.22	100.00 ± 6.30
	7.5	98.74 ± 7.30	108.35 ± 9.20	165.23 ± 9.80*
	15.0	89.78 ± 3.50*	105.30 ± 6.56	198.27 ± 9.30*
	22.5	78.27 ± 4.20*	96.25 ± 5.80	163.56 ± 7.60*

Experimental conditions were as described in Section 2. For each time of treatment the activity value obtained in the absence of **C1 **was considered as the control value (100%). *Significant differences (*P* > 0.05) were found when compared to the control as assessed by Student' *t* test.

## Abbreviations

Bnz: Benznidazole
APx: Ascorbate peroxidase
CPRG: Chlorophenol Red-*β*-D-galactopyranoside
DCF: Dichlorofluorescein
H_2_DCFDA: 2′,7′-Dichlorodihydrofluorescein diacetate
DIOC_6_: 3,3′-Dihexyloxacarbocyanine iodide
DTT: Dithiothreitol
FCCP: Trifluoromethoxy carbonyl cyanide phenyl hydrazone
FITC: Fluorescein isothiocyanate
GSH: Reduced glutathione
PI: Propidium iodide
PS: Phosphatidylserine
ROS: Reactive oxygen species
SOD: Superoxide dismutase
TCA: Trichloroacetic acid
Try: Trypanothione
TryR: Trypanothione reductase.
